# What should be our approach to knowledge translation in primary care?

**DOI:** 10.1186/1710-1492-6-S4-A4

**Published:** 2010-12-10

**Authors:** Samir Gupta

**Affiliations:** 1 Department of Medicine, University of Toronto, Toronto, Ontario, M5S 1A1, Canada

## 

Evaluations of delivered care have consistently demonstrated gaps between existing medical knowledge and current practice. Studies examining the quality of care across disciplines and jurisdictions have estimated that 30% to 45% of patients are not offered evidence-based best care, and 20% to 30% receive contraindicated and potentially harmful care [1,2].

Respiratory disease care gaps are equally alarming, with only 48% of recommended care delivered to patients with asthma exacerbations, and only 46% of routine recommended care delivered to patients with COPD [3]. Knowledge translation (KT) is a methodological approach developed specifically to address these care gaps. Because the most prevalent conditions are managed predominantly in primary care, there has been a great deal of interest in developing KT interventions targeting the primary care environment. A meta-analysis of randomized trials of guideline implementation interventions has demonstrated only modest effects on care across a wide range of disciplines, care settings, recommendation types and intervention types [4].

However, few of these studies employed behavioral theories to inform intervention design, and this lack of an appropriate theoretical underpinning may be partly responsible for their limited success [5]. A multi-step approach to KT intervention design might improve success. Knowledge implementers should start by investigating the theory-based factors that underlie existing clinical practice, in order to identify the theoretical constructs that should be targeted by an intervention. Once these factors are known, one can design interventions to enhance the processes supporting change in these specific constructs [6].

When targeting individual behaviour change, relevant theoretical categories include **motivational theories** (which explain how individuals wish, intend and ultimately decide to change behaviour), **action theories** (which explain how individuals move from intention to actual behaviour change), and **stage theories** (which describe an orderly progression through discrete stages toward behaviour change) [6]. Concepts of behavioural intention and self-efficacy are among the best predictors of subsequent health behaviour and are found in virtually all social cognitive models of health behaviour. Baseline factors influencing current behaviour can be identified directly through previous literature, or through direct measurement *via* interviews, questionnaires or group methods. An alternative approach is to analyze practice variation with respect to its determinants. With this technique, determinants identified predominantly in practices which adhere to or do not adhere to behaviour can be characterized as facilitators or barriers, respectively. Finally, researchers can analyze previously effective KT interventions to retrospectively ascertain which factors were likely influencing behaviour [7].

After having thus identified the relevant components of a behavior that should be targeted, an appropriate intervention can be developed based on approaches previously shown to be effective in other settings. General categories of interventions include educational interventions, which can be passive or interactive; audit-and-feedback; provision of “just-in-time” information, including reminders and clinical decision support systems; organizational changes such as role revisions or financial incentives; and patient-directed interventions such as pre-consultation questionnaires. Electronic tools are emerging as a modality to facilitate a wide range of these interventions and require further study.
